# Real-Time Measurement of Intrarenal Pressure Using LithoVue™ Elite: Focus on Small Ureteral Access Sheaths and Appropriate Irrigation Settings

**DOI:** 10.3390/jcm14103573

**Published:** 2025-05-20

**Authors:** Naoto Tanaka, Jose Carlo Elises, Fukashi Yamamichi, Yasuhiro Kaku, Yosuke Fukiishi, Masaichiro Fujita, Takaaki Inoue

**Affiliations:** 1 Department of Urology, Hara Genitourinary Hospital, Kobe City 650-0012, Hyogo, Japan; naoto.t501@gmail.com (N.T.); fukashi@train.ocn.ne.jp (F.Y.); superkaku7@gmail.com (Y.K.); yosukefukifuki@gmail.com (Y.F.); 0213masamasa@gmail.com (M.F.); 2 Division of Urology, Philippine General Hospital, Manila 1000, Philippines; carlo.elises@gmail.com; 3 Division of Urology, Department of Surgery Related, Kobe University Graduate School of Medicine, Kobe City 650-0017, Hyogo, Japan

**Keywords:** intrarenal pressure, LithoVue™ Elite, small ureteral access sheath, ratio of endoscope–sheath diameter, porcine kidney model

## Abstract

**Background/Objectives**: Intrarenal pressure (IRP) plays a critical role in ensuring the safety of retrograde intrarenal surgery (RIRS), as elevated IRP is associated with complications such as pyelovenous backflow, infection, and renal injury. LithoVue™ Elite (LVE) is the first commercially available ureteroscope (URS) capable of providing real-time IRP measurements. Conventionally, IRP has been measured via a percutaneous nephrostomy catheter (PNC), which may not accurately reflect dynamic changes during endoscopic procedures. Recently, small ureteral access sheaths (UASs) have been increasingly used to minimize ureteral injury risk. This study aimed (1) to assess the accuracy of LVE compared with that of IRP measured by a PNC and (2) to evaluate appropriate irrigation settings suitable for small UASs using porcine kidney models and LVE. **Methods**: An 11/13-Fr UAS and a 10/12-Fr UAS were inserted into each model, and an automatic irrigation pump (AIP) and hand pumping (HP) with a 20-cc syringe were used. IRP was measured at various LVE tip positions (renal pelvis and upper, middle, and lower calyces) with different irrigation settings, repeated four times in each. Simultaneously, the IRP via the PNC located in the upper calyx and renal pelvis was measured. **Results**: LVE showed high concordance with the PNC across the upper, middle, and lower calyces (*p* > 0.05). However, at the renal pelvis, LVE measured IRP values that were significantly higher than the PNC by a mean of 1.93 ± 0.93 mmHg (*p* < 0.001). For the 11/13-Fr UAS, the IRP remained below 30 mmHg across all irrigation settings with an AIP and HP. In contrast, the 10/12-Fr UAS maintained 30 mmHg only with limited AIP settings, while HP resulted in high IRP, exceeding 100 mmHg at any location. Intergroup comparisons demonstrated that the IRP with the 10/12-Fr UAS was significantly higher than that with the 11/13-Fr UAS at any irrigation pressure setting across all URS tip positions (*p* < 0.05). Intragroup comparisons indicated a significant pressure difference between the upper, middle, and lower calyces and the renal pelvis in both models at all irrigation settings (*p* < 0.05). **Conclusions**: LVE provided accurate IRP measurements compared to the PNC. The IRP was significantly influenced by UAS size, irrigation setting, and URS tip position. When using small UASs, selecting appropriate irrigation settings is essential to maintain the safe threshold.

## 1. Introduction

Retrograde intrarenal surgery (RIRS) is recognized as a standard treatment for renal stones up to 2 cm in size [1,2]. During RIRS, a ureteral access sheath (UAS) is frequently used to maintain a clear endoscopic view and adequate irrigation flow. Although the use of a UAS reduces intrarenal pressure (IRP) [3,4], elevated IRP during RIRS remains a major concern for urologists due to intra- and postoperative complications such as infection [5,6], pyelovenous backflow [5,6,7], forniceal rupture [5,8], excessive fluid absorption [5,6,7,8], and permanent kidney damage [5,8,9]. The threshold for the occurrence of intrarenal and pyelovenous reflux has been identified as 30–60 mm Hg [5,10,11,12]. Conventionally, IRP has been calculated percutaneously using a nephrostomy catheter (PNC) in previous studies [9,12,13]. However, this method may not accurately reflect dynamic pressure changes during irrigation.

Recent technological advancements have introduced novel ureteroscopes (URSs), such as LithoVue™ Elite (LVE; Boston Scientific, Marlborough, MA, USA), which incorporates a built-in pressure sensor. LVE is the first commercially available URS capable of providing real-time IRP, although few studies have measured IRP retrogradely using a URS [14,15,16,17,18]. Alongside device innovation, surgical practice has increasingly embraced smaller UASs to minimize the risk of ureteral injury with high insertion success rates [13,19,20]. However, the adoption of smaller UASs raises concerns about compromised irrigation efficiency and increased IRP owing to the restricted outflow pathway. Currently, limited data are available on IRP in the context of small UASs, and direct comparisons between newer pressure-sensing technologies and PNC-based measurements remain insufficient.

This study aimed (1) to assess the accuracy of LVE compared with that of IRP measured by a PNC and (2) to evaluate appropriate irrigation settings suitable for small UASs using porcine kidney models and LVE.

## 2. Materials and Methods

### 2.1. Experimental Setting

Fresh adult porcine perinephric tissue blocks and esophagi were obtained on the day of the experiment. The kidneys and upper ureters were removed en bloc, and the renal capsules were preserved. In one porcine model, each esophagus was continuously sutured to the distal end of the ureter to compensate for the length and small diameter of the whole ureter. The renal collecting system was inspected via URS to confirm the normal renal anatomy. The renal pelvis and upper, middle, and lower calyces were identified.

**Figure 1:**
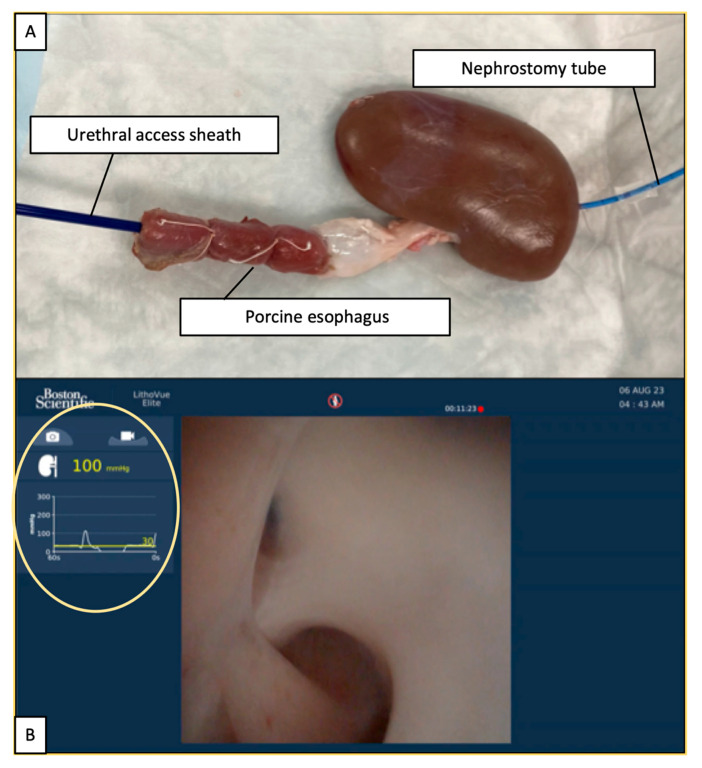
Experimental setting and LithoVue™ Elite image. (**A**) Ex vivo porcine kidney model. (**B**) The yellow circle shows real-time intrarenal pressure.

The posterior superior calyx was identified, and an 8.3-Fr PNC was inserted. The distal end of the PNC was placed inside the renal pelvis, making a loop. Thereafter, PNC was anchored to the renal parenchyma by sutures, and the proximal end of the PNC was connected to an arterial pressure monitor. A UAS was subsequently inserted with the distal tip just below the ureteropelvic junction (UPJ). Fluoroscopy was performed to confirm the position of the URS tip. A suture was used to secure the ureter and UAS together in order to maintain the UAS position and avoid irrigation fluid leakage (Figure 1A). Two kidney models were prepared, one with an 11/13-Fr UAS (Navigator^TM^ HD; Boston Scientific) and the other with a 10/12-Fr UAS (UroPass^®^; Olympus, Tokyo, Japan).

The renal collecting system was adequately distended using an automatic irrigation pump (AIP; UROMAT E.A.S.I.^®^; Karl Storz, Tuttlingen, Germany) with a setting of 100 mmHg for 1 min to soften the kidney, and no leakage was observed at the anastomotic and PNC sites.

### 2.2. Ureteroscope and IRP Measurements

LVE is a 9.5-Fr single-use flexible URS capable of measuring real-time IRP. The 7.7-Fr distal tip has a pressure sensor on the lateral side, and the IRP was assessed four times per second. LVE was calibrated before each procedure by immersing the tip in a saline solution to establish a baseline of 0 mmHg. The IRP was displayed on the same screen as in the endoscopic view (Figure 1B).

IRP was measured at various URS tip positions (renal pelvis and upper, middle, and lower calyces) with different irrigation settings and repeated four times. Two types of irrigation devices, an AIP and hand pumping (HP) with a 20-cc syringe, were used. To prevent sensor interference, the LVE tip was kept from contacting the mucosa throughout the procedures. The AIP settings ranged from 0 to 200 mmHg in 20 mmHg increments using saline. For the AIP, the maximum pressure value was recorded after 15 s of irrigation at the time of reaching its plateau. For the HP, the maximum pressure value was recorded after 3 s of flushing 10 mL of saline under 100 mmHg of irrigation pressure by the AIP. To minimize the influence of uncontrolled pressure spikes, saline was injected at a consistent speed. Simultaneously, the IRP measurements from the PNC were also recorded. To prevent model perforation, the IRP using HP was recorded at the end of the study. In this study, the safe threshold pressure was set at 30 mmHg, based on accepted standards for preventing pyelovenous backflow [9,10,11,12].

### 2.3. Statistical Analyses

Statistical analysis was performed using R version 4.1.1 (R Foundation, Vienna, Austria) and SPSS version 23.0 (IBM Corp., Armonk, NY, USA), with statistical significance set at a *p* value of <0.05. The accuracy of LVE and the PNC was compared using a mixed repeated measures analysis of variance. To examine the appropriate pressure settings for each UAS, the Mann–Whitney test was used for intergroup comparisons, whereas the Kruskal–Wallis test was used for intragroup comparisons for the skewed distribution of variances.

## 3. Results

**Figure 2:**
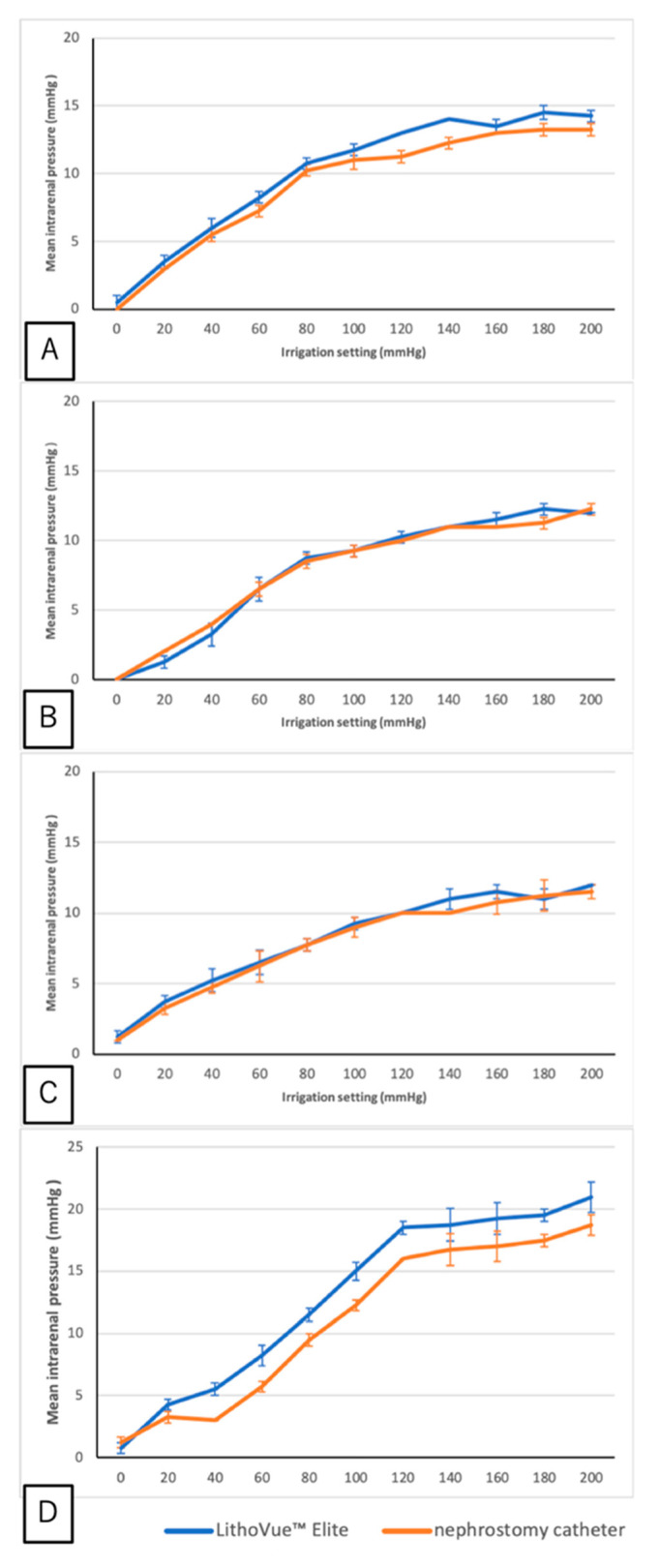
Comparison of intrarenal pressure (IRP) between LithoVue™ Elite (LVE) and nephrostomy catheter (PNC) in each calyx and renal pelvis. (**A**) LVE tip in the upper calyx. (**B**) LVE tip in the middle calyx. (**C**) LVE tip in the lower calyx. (**D**) LVE tip in the renal pelvis. (**A**–**C**) No significant difference was observed between the two devices in any calyx. (**D**) In the renal pelvis, the mean IRP measured by LVE was higher (1.93 ± 0.93 mmHg) than that measured by the PNC (*p* < 0.001).

All measurements were successfully recorded without any signs of leakage. For accuracy, the IRP values measured by LVE and the PNC increased in parallel with similar trends as the AIP settings increased when the LVE tip was at the upper, middle, and lower calyces (*p* = 0.057, *p* = 0.780, and *p* = 0.103, respectively). However, when the tip was positioned at the renal pelvis, the mean IRP measured by LVE was higher by 1.93 ± 0.93 mmHg than that of the PNC (*p* < 0.001) (Figure 2).

**Figure 3:**
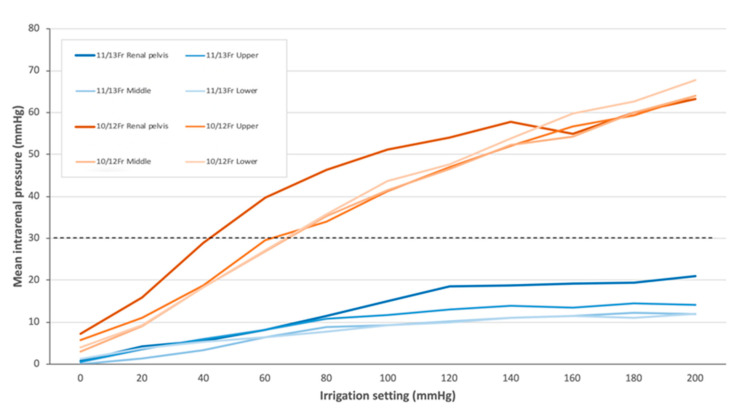
Linear graph showing the intrarenal pressure (IRP) at eight locations for two ureteral access sheaths (UASs) when using an automatic irrigation pump (AIP).

**Figure 4:**
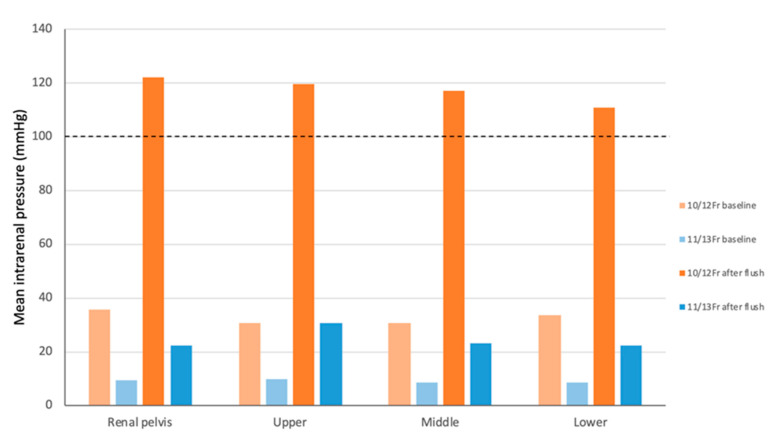
Bar graph showing the intrarenal pressure (IRP) at eight locations for two ureteral access sheaths (UASs) when using hand pumping (HP).

The IRP measured by LVE is shown in Table 1. For AIP, with the 11/13-Fr UAS, IRP remained below 30 mmHg at all irrigation settings at any URS tip location; with the 10/12-Fr UAS, IRP was below 30 mmHg at irrigation pressures of 0, 20, and 40 mmHg in the renal pelvis and at 0, 20, 40, and 60 mmHg in any calyx (Figure 3). Regarding HP, with the 11/13-Fr UAS, IRP was below 30 mmHg at almost all locations, except for the upper calyx (mean value, 30.75 mmHg); with the 10/12-Fr UAS, the IRP exceeded 100 mmHg at any location (Figure 4).

Intergroup comparisons demonstrated that the IRP with the 10/12-Fr UAS was significantly higher than that with the 11/13-Fr UAS at any irrigation pressure setting across all URS tip positions (*p* < 0.05). Intragroup comparisons indicated a significant pressure difference between the upper, middle, and lower calyces and the renal pelvis in both models at all irrigation settings (*p* < 0.05).

## 4. Discussion

The purpose of the present study was to assess the accuracy of LVE compared with that of a PNC in measuring IRP and to evaluate appropriate irrigation settings suitable for small UASs. To the best of our knowledge, this is the first study to compare the accuracy between LVE and a PNC, which has been commonly used in previous studies for IRP measurement, and to report on appropriate pressure settings, particularly for small UASs. Our findings indicate that LVE provides accurate real-time IRP. Furthermore, our study identifies the appropriate irrigation settings for small UASs.

Elevated IRP during RIRS is a well-recognized factor that contributes to both immediate and long-term adverse outcomes. Complications such as pyelovenous and pyelolymphatic backflow, postoperative fever, systemic infections, and even renal parenchymal damage have been reported to be associated with elevated IRP during endourological interventions [5,6,7,8,9,10,11,12]. Previous studies have primarily evaluated IRP using pressure sensors or transducers via a urethral catheter or a PNC placed in the renal pelvis [9,12,13]. Hence, the measured values may not always reflect actual conditions during RIRS. Recent technological advances, such as LVE, fiber optic pressure sensors [17,18], and UASs integrating irrigation, aspiration, and IRP measurements [21] now allow IRP assessments similar to actual RIRS conditions reflecting dynamic pressure changes. Advances have also produced thinner devices, increasing the importance of small UASs in minimizing the risk of ureteral injury [13,19,20].

LVE demonstrated high-accuracy data compared with the PNC across all calyces. When positioned in the renal pelvis, LVE revealed a mean IRP of 1.93 ± 0.93 mmHg, higher than that of the PNC. The pressure sensor located on the lateral side of LVE may interfere with the PNC. In contrast, the PNC has multiple holes at its tip, thus minimizing the interference. Another study on a bench model with LVE showed at least 96% full-scale accuracy [15]. The fiber optic pressure sensors that can be inserted through the working channel of a URS may mitigate the interference issues.

To maintain a low IRP and an acceptable flow rate, the concept of the ratio of endoscope–sheath diameter (RESD) is gaining attention. The RESD is defined as the ratio of the outer diameter of a URS to the inner diameter of a UAS. A higher RESD may elevate the intrarenal resistance and IRP. Although the ideal RESD has not been established, some studies suggest that the RESD should be <0.75 when using a UAS without negative pressure suction to maintain safe thresholds [22]. In this study, the RESD was 0.95 for the 10/12-Fr UAS and 0.86 for the 11/13-Fr UAS. Although these values exceed the suggested threshold, the advantage of LVE is that the irrigation settings can be adjusted based on real-time IRP information, leading to safer irrigation strategies during RIRS. Sener et al. highlighted the compatibility between URSs and UASs and emphasized the advantages of the 10/12-Fr UAS in optimally balancing between ureteral wall impact and IRP [19]. Larger UASs may be necessary when using LVE without concerns about irrigation; however, Traxer et al. reported a potential risk of severe injuries to the ureter muscular layer with UAS sizes of 12/14 Fr and above [23]. Our study demonstrates the importance of careful pressure adjustment, particularly with the 10/12-Fr UAS, where HP poses risks. In contrast, the 11/13-Fr UAS allowed a wide range of irrigation settings while maintaining the safe pressure threshold. However, excessive irrigation still increases the IRP. Wilson et al. showed that forceful manual irrigation with a 60-cc syringe could raise the pressure up to 440 mmHg [24]. Vergamini et al. revealed that HP generated significantly higher IRP than the AIP when measured by LVE [14].

Despite its growing clinical use, only limited data are available for the 10/12-Fr UAS, owing to infrequent use in other studies. Bhojani et al. reported significantly higher IRP with the 10/12-Fr UAS compared to larger UASs during in vivo RIRS using LVE, which was attributed to reduced irrigation outflow. In their study, 14% of all patients received the 10/12-Fr UAS [16]. Another study using a retrogradely placed vascular pressure wire with gravity irrigation at 40 cmH_2_O reported baseline IRP values ranging from 13.14 to 33.99 cmH_2_O with the 10/12-Fr UAS during RIRS in 14 patients [25], consistent with our data. Fang et al. revealed that with a combination of the 9.9-Fr URS and 11/13-Fr UAS, IRP exceeded 40 cmH_2_O at irrigation pressures of 250 cmH_2_O [22], supporting our data. A unit of mmHg can be converted to cmH_2_O based on a conversion calculation of 1 cmH_2_O = 0.736 mmHg.

Additionally, the URS tip position has been shown to affect IRP; however, the impact remains controversial, and the influence of the PNC located at the upper calyx and renal pelvis cannot be overlooked. Our study demonstrates a significant pressure difference among the four locations, although multiple comparisons could not reveal the differences owing to the limited sample size. When the URS tip was positioned in the renal pelvis, the IRP was higher than that in any calyx for both UASs. With the URS tip in the upper calyx and using the 11/13-Fr UAS with HP, the IRP was marginally higher. Patel et al. found that the IRP in the calyx exceeded that in the renal pelvis, suggesting that infundibular dimensions may influence this phenomenon [26]. In contrast, Guan et al. reported no significant IRP differences between the calyces and the renal pelvis [27]. However, similar to our findings, Bai et al. identified significant IRP differences between the upper, middle, and lower renal calyces and the UPJ, with IRP in the UPJ being higher under certain conditions [17]. Jung et al. also reported higher IRP when the URS tip was in the upper calyx during supine minimally invasive percutaneous nephrolithotomy [28], supporting our findings. These results collectively demonstrate that the position of the URS tip affects IRP, although anatomical factors have not been thoroughly analyzed.

Several limitations of the present study should be acknowledged. First, this study used ex vivo porcine kidneys, which closely resemble human kidneys in terms of the size, anatomy, and tissue composition; however, these ex vivo models lacked the dynamic response of IRP changes in the upper urinary tract. Second, the potential influence of the PNC, located in the upper calyx and renal pelvis should be considered owing to its effects on fluid dynamics [12]. Third, the study limited the UAS tip positioning to the UPJ, which, although commonly used, does not reflect the variability in UAS placement observed in clinical practice. Forth, simulated RIRS was not performed with the working channel occupied by laser fibers or baskets. Additionally, the limited number of experimental repetitions (four per configuration) reduced the statistical power of the findings. Only two UAS sizes were evaluated (10/12-Fr and 11/13-Fr), selected to reflect the increasing clinical preference for smaller UASs to minimize ureteral injury. Future ex vivo studies should incorporate larger UASs (e.g., 12/14-Fr or 14/16-Fr) to provide a more comprehensive understanding of the IRP. Finally, the irrigation flow rates were not quantified. Given that the flow rate directly influences IRP, future studies should investigate the relationship between irrigation flow and IRP to optimize endoscopic safety and performance.

In summary, this study demonstrated the accuracy of LVE and identified appropriate irrigation settings for the 10/12-Fr and 11/13-Fr UASs with LVE using porcine kidney models. In addition, the factors influencing IRP were identified. LVE enables urologists to easily obtain real-time IRP information without the need for additional devices. When using small UASs, a controlled AIP is preferable to HP. Further investigations on IRP with small UASs are warranted to improve safe procedures.

## 5. Conclusions

LVE provided accurate IRP measurements, with results comparable to those of previous studies. IRP was significantly influenced by UAS size, irrigation settings, and URS tip position. This study highlights the importance of appropriate irrigation settings for smaller UASs. Notably, precise pressure adjustments are essential when using an AIP, while excessive inflow via HP results in an extremely high IRP. Compared with larger UASs, small UASs are disadvantageous for maintaining a lower IRP; however, they offer the advantage of reducing the risk of ureteral injury. Appropriate irrigation settings compensate for the disadvantage and help ensure safe RIRS. Easy access to real-time IRP can provide critical insights that enhance the safety of lithotripsy procedures and prevent complications.

## Figures and Tables

**Table 1:** Summary of intrarenal pressures with different irrigation settings.

	Summary of Intrarenal Pressure (Mean ± SD, mmHg)													
Irrigation setting		Automatic irrigation pump											Hand pumping	
		0 mmHg	20 mmHg	40 mmHg	60 mmHg	80 mmHg	100 mmHg	120 mmHg	140 mmHg	160 mmHg	180 mmHg	200 mmHg	Baseline	After flush
11/13-Fr	Upper pole	0.5 ± 0.50	3.5 ± 0.50	6 ± 0.70	8.25 ± 0.43	10.75 ± 0.43	11.75 ± 0.43	13 ± 0	14 ± 0	13.5 ± 0.50	14.5 ± 0.50	14.25 ± 0.43	9.75 ± 0.43	30.75 ± 2.49
	Middle pole	0 ± 0	1.25 ± 0.43	3.25 ± 0.83	6.5 ± 0.87	8.75 ± 0.43	9.25 ± 0.43	10.25 ± 0.43	11 ± 0	11.5 ± 0.50	12.25 ± 0.43	12 ± 0	8.75 ± 0.43	23.25 ± 0.43
	Lower pole	1.25 ± 0.43	3.75 ± 0.43	5.25 ± 0.83	6.5 ± 0.87	7.75 ± 0.43	9.25 ± 0.43	10 ± 0	11 ± 0.71	11.5 ± 0.50	11 ± 0.71	12 ± 0	8.75 ± 0.43	22.5 ± 0.50
	Renal pelvis	0.75 ± 0.43	4.25 ± 0.43	5.5 ± 0.50	8.25 ± 0.83	11.5 ± 0.50	15 ± 0.71	18.5 ± 0.50	18.75 ± 1.30	19.25 ± 1.30	19.5 ± 0.50	21 ± 1.22	9.5 ± 0.87	22.5 ± 0.87
10/12-Fr	Upper pole	5.75 ± 0.43	11 ± 0.71	18.75 ± 1.92	29.5 ± 1.11	34 ± 0.71	41.25 ± 1.64	47 ± 1.22	52 ± 0.71	56.75 ± 1.09	59.25 ± 0.83	64 ± 1.87	30.75 ± 0.83	119.75 ± 1.79
	Middle pole	3 ± 0.71	9 ± 0	18.25 ± 0.43	27 ± 0	35.25 ± 0.43	41.5 ± 0.50	46.5 ± 1.12	52.25 ± 0.83	54.25 ± 1.30	60 ± 0	64 ± 0.71	30.75 ± 0.43	117.25 ± 7.89
	Lower pole	4 ± 1.00	9.25 ± 0.43	18.25 ± 0.43	27.25 ± 1.09	35.75 ± 0.83	43.75 ± 0.43	47.75 ± 0.83	53.75 ± 1.09	59.75 ± 1.48	62.75 ± 0.83	67.75 ± 0.83	33.75 ± 0.83	110.75 ± 3.11
	Renal pelvis	7.25 ± 1.09	16 ± 0.71	29 ± 0	39.75 ± 1.09	46.25 ± 1.64	51.25 ± 0.83	54 ± 1.22	57.75 ± 1.48	55 ± 0.71	60 ± 0	63.25 ± 1.92	35.75 ± 0.83	122.25 ± 7.01

## Data Availability

The original contributions presented in this study are included in the article. Further inquiries can be directed to the corresponding author(s).
